# Effect of dimethyl sulfoxide on universal adhesives resin bonding to caries-affected dentine

**DOI:** 10.1007/s00784-026-07258-z

**Published:** 2026-11-14

**Authors:** Camila Farias Monteles, Michel Wendlinger, Gabriel D. Cochinski, Thiago Henrique Scarabello Stape, Diego Melendez, Arzu Tezvergil-Mutluay, Alessandro D. Loguercio

**Affiliations:** 1 Department of Restorative Dentistry, School of Dentistry, State University of Ponta Grossa, Rua Carlos Cavalcanti, 4748 Bloco M, Sala 64-A, Uvaranas, Ponta Grossa, Paraná 84030-900 Brazil; 2 Department of Restorative Dentistry and Cariology, Adhesive Dentistry Research Group, Institute of Dentistry, University of Turku, Turku, Finland; 3 Turku University Hospital, TYKS, University of Turku, Turku, Finland; 4 Department of Restorative Dentistry, Facultad de Odontología, Universidad de los Andes, Santiago, Chile

**Keywords:** Caries-affected dentine, Dimethyl sulfoxide, Dentin bonding, Universal adhesives, Adhesive interface

## Abstract

**Objectives:**

To evaluate the effect of dimethyl sulfoxide (DMSO) on the microtensile bond strength (µTBS), nanoleakage (NL), and degree of conversion (DC) of universal adhesives applied to sound and caries-affected dentine.

**Materials and methods:**

One hundred and twenty-eight human molars were assigned to 16 groups according to dentine substrate (sound or caries-affected), adhesive system (ZipBond Universal [ZIP] or Tetric N-Bond Universal [TNU]), application mode (etch-and-rinse or self-etch), and pretreatment (with or without 50% DMSO). Composite resin restorations were placed, and resin-dentine sticks were prepared for µTBS, NL, and DC analyses. Data were analyzed using four-way ANOVA, Tukey’s test (α = 0.05), and Weibull analysis.

**Results:**

DMSO pretreatment significantly affected the bonding performance of both universal adhesives. Overall, sound dentine showed higher µTBS and DC values and lower NL than caries-affected dentine (*p* < 0.05). Caries-affected dentine without DMSO exhibited the lowest µTBS, DC, Weibull parameters, and the highest NL values (*p* < 0.05). DMSO significantly increased µTBS, DC, Weibull modulus, and characteristic strength while reducing NL, with the greatest improvements observed in caries-affected dentine (*p* < 0.05). No significant differences were found between etch-and-rinse and self-etch approaches (*p* > 0.05).

**Conclusion:**

DMSO pretreatment improved the bonding performance, interfacial integrity, polymerization, and reliability of universal adhesives, particularly when applied to caries-affected dentine.

## Introduction

Adhesive dentistry has evolved substantially over recent decades, enabling minimally invasive restorative approaches and preservation of dental tissues [1]. However, the longevity of adhesive restorations remains highly dependent on the quality and durability of the resin-dentine interface [2, 3]. Bonding to dentine is intrinsically more complex than bonding to enamel because of its heterogeneous composition, intrinsic moisture, tubular structure, and organic matrix-rich nature [4, 5]. These challenges become even more pronounced when adhesion is performed on altered substrates, such as caries-affected dentine [6–8].

Caries-affected dentine (CAD) is a partially demineralized substrate that is commonly preserved during minimally invasive caries removal procedures [9]. Compared with sound dentine, CAD presents significant structural and chemical alterations, including reduced mineral content, increased porosity, occluded dentinal tubules, disorganized collagen fibrils, and higher intrinsic water content [10–12]. These changes compromise the mechanical properties and ultrastructural integrity of the substrate, negatively affecting adhesive infiltration and hybrid layer formation [13]. Moreover, altered dentine permeability and the reduced availability of stable mineral phases may impair the chemical interaction between functional adhesive monomers and dentine [14, 15], ultimately compromising bond strength and long-term interfacial stability.

Several strategies have been proposed to improve the durability of resin bonding to CAD, including the use of silver diamine fluoride [16, 17], matrix metalloproteinase inhibitors [18], antimicrobial nanoparticles [19, 20], and biomodification agents [21, 22]. Among these approaches, dimethyl sulfoxide (DMSO) has attracted increasing attention because of its unique physicochemical properties [23]. DMSO is a polar aprotic solvent with high penetration ability and affinity for both hydrophilic and hydrophobic molecules [24]. Owing to its amphiphilic nature and strong interaction with water molecules, DMSO can reduce water self-association and improve dentine wettability [25]. These characteristics make DMSO a promising agent for improving the resin-dentine bond. Previous studies have demonstrated that DMSO pretreatment may improve resin monomer infiltration, reduce residual water within the hybrid layer, and enhance bond stability over time [26–28]. However, most of these studies have focused on sound dentine.

Recently, Ismail et al. (2026) [29] assessed the influence of DMSO pretreatment on cervical carious lesions and demonstrated that DMSO was effective in improving bonding to this substrate. However, in that study, DMSO was applied at a low concentration (10%) and exclusively in association with a two-step etch-and-rinse adhesive system. Since 50% DMSO has been more commonly investigated because of its ability to enhance dentinal collagen expansion and facilitate deeper monomer infiltration [30], its application to CAD may provide greater benefits for adhesive performance. Furthermore, the available evidence remains limited because only one adhesive strategy has been evaluated in carious dentine. Investigating the interaction between DMSO pretreatment and universal adhesives is particularly relevant because these materials rely on functional monomers, such as methacryloyloxydecyl dihydrogen phosphate (10-MDP), to establish chemical interactions with calcium [14]. In CAD, where mineral content is reduced and the substrate is structurally altered, the interaction among DMSO, residual calcium availability, and 10-MDP-mediated bonding may differ substantially from that observed in sound dentine [31, 32]. Therefore, understanding the behavior of DMSO when combined with universal adhesive systems may provide important insights into the performance and effectiveness of adhesion to CAD.

Thus, this study aimed to evaluate the effect of DMSO pretreatment on resin bonding to sound and caries-affected dentine, using two universal adhesives applied in the etch-and-rinse or self-etch mode. Three null hypotheses were tested: (i) DMSO pretreatment would not affect resin bond strength, irrespective of dentine substrate, adhesive, or application mode; (ii) DMSO pretreatment would not affect nanoleakage, irrespective of dentine substrate, adhesive, or application mode; and (iii) DMSO pretreatment would not affect the degree of conversion, irrespective of dentine substrate, adhesive, or application mode.

## Materials and methods

### Experimental design

The present study was designed to evaluate four study factors: (1) dentine substrate, with two levels (sound and caries-affected); (2) adhesive system, with two levels (ZipBond Universal [ZIP] and Tetric N-Bond Universal [TNU]); (3) application mode, with two levels (etch-and-rinse and self-etch); and (4) treatment, with two levels (untreated and 50% dimethyl sulfoxide [DMSO 50%]). A total of 128 extracted caries-free human molars were randomly allocated into 16 experimental groups (*n* = 8) according to the combination of all study factors.

### Selection and teeth preparation

A total of 128 extracted caries-free human molars were used in this study. The teeth were collected after the patients provided informed consent and approval was obtained from the Ethics Committee of the State University of Ponta Grossa, PR, Brazil (protocol #7.205.003). The teeth were disinfected in 0.5% chloramine, and the occlusal dentine surface was exposed by removing the occlusal third of the crown using a diamond saw (Isomet, Buehler; Lake Bluff, IL, USA) under water cooling to obtain a flat dentine surface.

### Production of artificial dentine caries

Except for the sound dentine groups, the teeth were subjected to an artificial dentine caries procedure as described in previous studies [16, 33, 34]. The sound dentine specimens were stored in distilled water at 37 °C until testing. The teeth assigned to the caries-affected groups were initially sealed with a layer of epoxy adhesive (Ciba Espec Quim, São Paulo, SP, Brazil), followed by a layer of nail polish, leaving the occlusal dentine surface exposed.

The specimens were then immersed in a cariogenic medium consisting of 3.7 g of BHI (Becton Dickinson, Sparks, MD, USA), 2 g of sucrose, 1 g of glucose, and 0.5 g of yeast extract (Sigma-Aldrich, St. Louis, MO, USA) in 100 mL of distilled water. The solution was sterilized at 121 °C for 20 min in an autoclave before being inoculated with 10^8 CFU/mL of Streptococcus mutans ATCC 25,175 (obtained from the National Institute of Quality Control in Health, Fiocruz, RJ, Brazil). The teeth were then incubated at 37 °C in a microaerophilic jar for 14 days. During this period, the cariogenic solution was replaced every 48 h without additional bacterial inoculation.

The biofilm was removed with gauze, and the insulating materials were manually removed using scalpel blades. The teeth were thoroughly washed with deionized water, and a darkened, softened dentine surface was identified using an exploratory probe [16, 33]. The outer softened carious dentine layer was removed by polishing with 600-grit silicon carbide paper for 20 s under light pressure. This procedure was performed only once to preserve the firm carious dentine lesion while yielding a flat dentine surface [16]. The endpoint for softened carious tissue removal was identified when the dark dentine surface firmly resisted a sharp excavator but required some pressure during manual removal [35]. This procedure was performed by a single calibrated and experienced operator who had been previously trained.

All teeth underwent a microhardness test before the demineralization process (sound dentine), immediately after induction of the caries lesion on the tooth surface (caries-infected dentine), and after removal of the outer softened carious dentine layer (caries-affected dentine) to confirm dentine demineralization. The microhardness test was performed using a Shimadzu HMV II microhardness tester (Kyoto, Japan) with a Knoop indenter. A static load of 25 g was applied for 30 s to sound dentine, whereas a 10-g load was applied for 30 s to carious dentine. The use of different loads was necessary because well-defined indentations with minimal surrounding fractures could not be reliably obtained in both types of tissue using the same load, given their distinct structural characteristics [36]. Five readings were obtained from each tooth, with a minimum spacing of 2.5 times the long diagonal of the indentation to avoid overlap between adjacent impressions, and averaged to determine the Knoop hardness of each sample [37]. Caries-affected dentine was determined until Knoop hardness reached approximately 50% of the initial values of sound dentin. All procedures were performed to standardize the creation of carious lesions in all teeth included in the study.

### Restorative procedures

The DMSO pretreatment solution was prepared by diluting DMSO (Sigma-Aldrich; St. Louis, MO, USA; pH 8.2) in distilled water to a final concentration of 50% (v/v), as previously described [27, 30]. DMSO pretreatment was applied after acid etching for the etch-and-rinse approach and before adhesive application for the self-etch approach. For both strategies, 50 µL of the water-based DMSO solution was scrubbed onto the dentine surface for 60 s using a microbrush (Cavibrush Blue, FGM Dental Group, Joinville, SC, Brazil). Excess solvent was removed by gently applying an air stream for 5 s using an air syringe positioned 10 cm from the dentine surface.

Regardless of DMSO application, the dentine surfaces were kept slightly moist before adhesive application in the etch-and-rinse groups, following the DMSO wet-bonding protocol [26]. For the self-etch approach, the dentine surfaces were kept slightly dry. Bonding procedures were performed in either etch-and-rinse or self-etch mode according to the manufacturers’ recommendations. Further details regarding adhesive application are presented in Table 1.

**Table 1:** Adhesives systems, batch number, composition and application modes

Adhesive system (Batch number)	pH	Composition	Application mode	
			Etch-and-rinse	Self-etch
Tetric N-Bond Universal (TNU, Ivoclar Vivadent, # ZO73H0)	2.5-3.0	HEMA, 10-MDP, Bis-GMA, MCAP, D3MA, ethanol, water, highly dispersed silicon dioxide and CQ	1. Apply etchant for 10–15 s. 2. Rinse for 10 s. 3. Air dry 5 s. 4. Apply adhesive as for the self-etch mode.	1. Applied one coat of adhesive to the entire preparation with a microbrush and rub it in for 20 s. 2. Gently air thinned for 5 s 3. Light-cured for 10 s at 1200 mW/ cm^2^
ZipBond (ZIP, SDI, #1232488)	2.5	10-MDP, UDMA, aliphatic dimethacrylate, hydrophilic aliphatic monomethacrylate, acidic monomethacrylate, ethanol (30–35%), butanone, water, initiator and fluoride.	1. Apply etchant 15 s 2. Rinse for 15 s 3. Air dry 2 s 4. Apply adhesive as for the self-etch mode	1. Apply the adhesive to the entire preparation with a microbrush and rub it in for 10 s. 2. Wait more 10 s 3. Evaporate excess solvent by air-drying for 5 s 4. Light cure for 10 s at 1200 mW/ cm^2^

*HEMA* 2-hydroxyethyl methacrylate, *10-MDP* methacryloyloxydecyl dihydrogen phosphate, *Bis-GMA* bisphenol A diglycidyl methacrylate, *MCAP* methacrylated carboxylic acid polymer, *D3MA* decandiol dimethadrylate, *CQ* camphorquinone, *UDMA* urethanedimethacrylate

After the bonding procedure, a composite resin restoration (Opallis, FGM Dental Group, Joinville, SC, Brazil) was applied in two or three increments, each 2 mm thick. Each increment was light-cured for 40 s at a distance of 1 mm using an LED curing unit set at 1,200 mW/cm² (Raddi, SDI Limited, Bayswater, Victoria, Australia). The teeth were then stored in distilled water at 37 °C for 24 h.

Subsequently, the teeth were sectioned into mesiodistal and buccolingual segments using a precision cutting machine (Isomet, Buehler; Lake Bluff, IL, USA) under water cooling to obtain resin-dentine bonded sticks with a cross-sectional area of approximately 0.8 mm², measured using a digital caliper (Digimatic Caliper, Mitutoyo, Tokyo, Japan). All resin-dentine bonded sticks were inspected under an optical microscope (SZH131, Olympus; Tokyo, Japan) at 40× magnification to detect voids or cracks in the bonded interface.

The resin-dentine bonded sticks obtained from each tooth, considering both the central and peripheral portions of the bonded interface for all experimental condition were allocated as follows: two sticks were used for nanoleakage (NL) evaluation, and two were used to assess the in situ degree of conversion (DC) within the adhesive/hybrid layers. These analyses are interface-focused, high-resolution techniques that require a limited number of specimens. The remaining sticks (a minimum of eight per tooth) were used for microtensile bond strength (µTBS) testing, which requires a larger number of specimens to account for interfacial variability and ensure adequate statistical reliability, in accordance with established methodological recommendations [38–40].

### Microtensile bond strength test (µTBS)

Microtensile bond strength testing was performed according to the Academy of Dental Materials guidelines for non-trimmed µTBS testing [38]. Resin-dentine bonded sticks were attached to a Geraldeli jig using cyanoacrylate adhesive and tested under tension (Kratos Dinamômetros, Cotia, SP, Brazil) at a crosshead speed of 0.5 mm/min until failure. µTBS values (MPa) were calculated by dividing the maximum load at failure (N) by the cross-sectional bonding area (mm²). The mean µTBS of the sticks obtained from the same tooth was calculated for statistical purposes.

Failure modes of the resin-dentine bonded sticks were classified as cohesive (C; failure exclusively within the dentine or composite resin) or adhesive/mixed (A/M; failure at the resin-dentine interface or failure at the resin-dentine interface associated with partial cohesive failure of the adjacent substrates). Failure modes were determined using a stereomicroscope at 100× magnification (Olympus SZ40, Tokyo, Japan). The number of premature failures (PF) was recorded but was not included in the calculation of the mean bond strength.

### Nanoleakage evaluation (%)

Resin-dentine bonded sticks were coated with two layers of nail varnish applied up to 1 mm from the bonded interfaces. The specimens were rehydrated in distilled water for 10 min before immersion in the silver tracer solution. Ammoniacal silver nitrate was prepared according to the protocol previously described by Tay et al. [39].

The resin-dentine bonded sticks selected for this test were immersed in a 50 wt% ammoniacal silver nitrate solution in complete darkness for 24 h. The specimens were then thoroughly rinsed with distilled water and immersed in a photo-developing solution for 8 h under fluorescent light to reduce silver ions to metallic silver grains within voids along the bonded interface.

The specimens were polished with 1000-, 1500-, 2000-, and 2500-grit silicon carbide paper and 1- and 0.25-µm diamond pastes (Buehler Ltd., Lake Bluff, IL, USA) and then mounted on aluminum stubs. The specimens were ultrasonically cleaned, air-dried overnight, and gold-sputtered (MED 010, Balzers Union, Balzers, Liechtenstein).

The interfaces were examined using a scanning electron microscope in backscattered electron mode at 15 kV (VEGA 3 TESCAN, Shimadzu, Tokyo, Japan). Three images were obtained from each specimen. The first image was acquired from the center of the resin-dentine bonded interface, whereas the other two were obtained 300 μm to the left and right of the first image. Images were acquired by a blinded, experienced operator. The relative percentage of NL within the adhesive and hybrid layer areas was measured in all images using ImageJ, a public-domain Java-based image-processing software package developed by the National Institutes of Health (NIH) [41].

### In situ degree of conversion (DC)

The resin-dentine bonded sticks were wet-polished, ultrasonically cleaned, and positioned in a micro-Raman microscope (XploRA ONE, HORIBA Scientific; Piscataway, NJ, USA), which was previously calibrated using a silicon standard. Samples were analyzed using a 532-nm diode laser through a 100× air objective. Raman spectra were acquired using a 600 lines/mm grating over a spectral range of 400–2000 cm⁻¹.

The acquisition settings included 100 mW laser power, 3-µm spatial resolution, 1-cm⁻¹ spectral resolution, and a 30-s accumulation time with five co-additions. Spectra were collected at three randomly selected sites within the hybrid layer of each stick. Post-processing was performed using Opus Spectroscopy Software (version 6.5, HORIBA Scientific; Piscataway, NJ, USA). Spectra obtained from uncured adhesives served as references.

DC was determined from the ratio of the aliphatic C = C peak at 1638 cm⁻¹ to the aromatic C = C peak at 1608 cm⁻¹ in cured and uncured samples (*n* = 5). DC was calculated using a previously published​ formula [40]. In addition, the most intense peaks and their corresponding chemical bonds were recorded for all materials.

### Statistical analysis

Sample size calculation was performed using a website designed for this purpose (http://www.sealedenvelope.com). Microtensile bond strength values for the tested mild universal adhesive (Tetric N-Bond Universal) bonded to caries-affected dentine were obtained from the literature [42]. Based on the range of µTBS differences reported in the literature for comparable experimental conditions [43], an expected between-group difference of 9 MPa was selected as the target effect for the sample size calculation. Using a significance level of 0.05 and 80% statistical power, the minimum required sample size was estimated at eight teeth per group (*n* = 8), accounting for potential specimen loss.

The mean µTBS (MPa), NL (%), and DC (%) values of the bonded sticks obtained from the same tooth were averaged for statistical purposes; thus, the tooth was considered the experimental unit. Premature failures were not included in the tooth mean for statistical analysis. After confirming normality of the data distribution using the Kolmogorov-Smirnov test and homogeneity of variances using Bartlett’s test, µTBS (MPa), NL (%), and DC (%) data were subjected to a four-way repeated-measures ANOVA, considering dentine, adhesive, bonding strategy, and treatment as factors. Tukey’s test was subsequently applied for post hoc comparisons. All analyses were performed at a significance level of 5% using Jamovi Statistical Software, version 2.3.

Microhardness (KHN) data were analyzed using a paired Student’s t-test. Furthermore, the reliability of the resin-dentine bonded interfaces was evaluated using Weibull analysis. Bond strength data from the resin-dentine sticks in each group were used to calculate the Weibull modulus (m) and characteristic strength (σ₀), with 95% confidence intervals. Weibull parameters were estimated using maximum likelihood estimation (MLE) and compared using the likelihood ratio test (LRT) (R Statistical Software, version 4.5.3).

## Results

### Microhardness test (KHN)

**Fig. 1:**
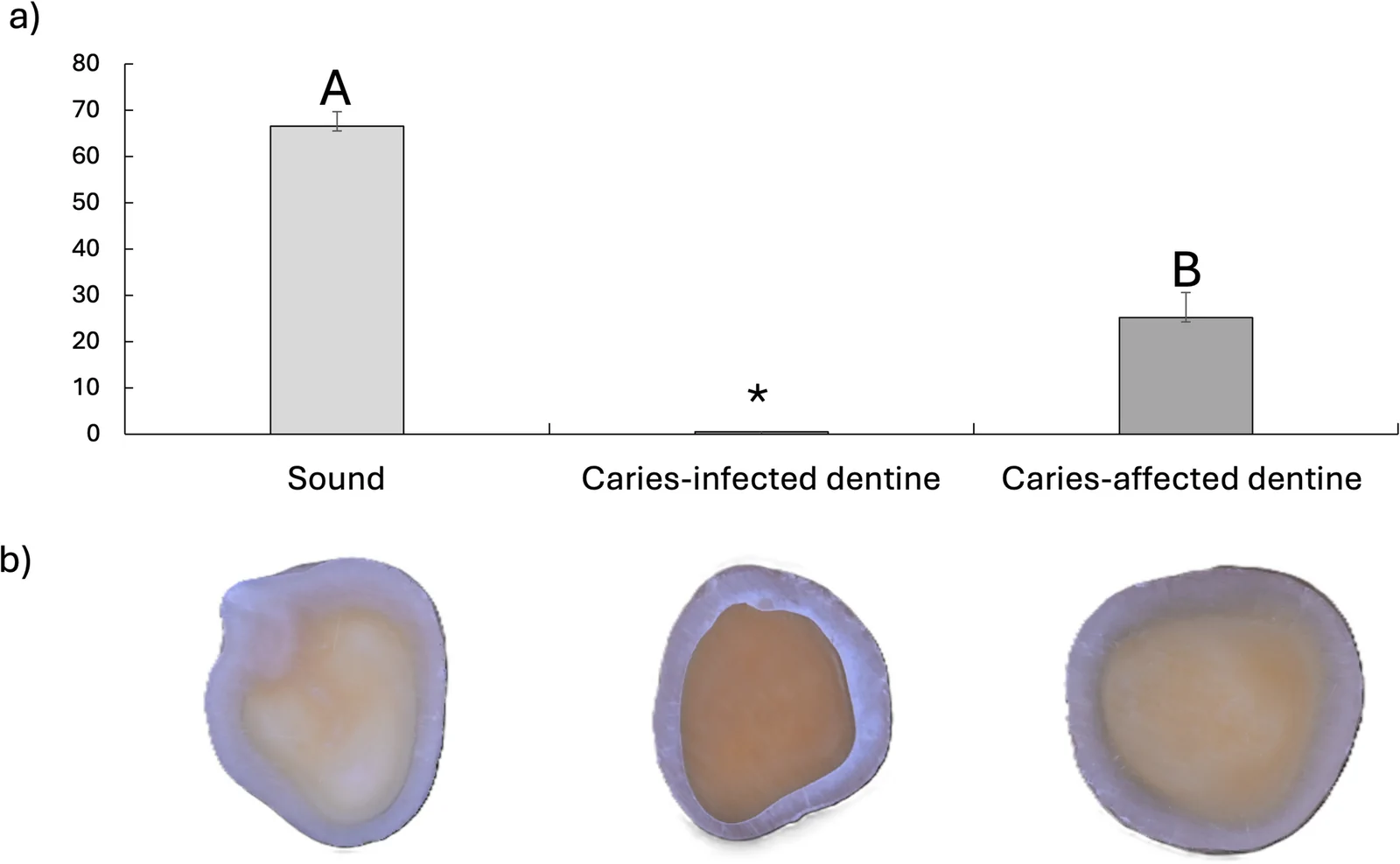
(**A**) Knoop microhardness (KHN) values (mean ± standard deviation) for the different substrates. Different capital letters indicate differences between groups (Paired Student’s t test; *p* < 0.001); (**B**) Representative images of sound, caries-infected and caries-affected dentine

The KHN values are presented in Fig. 1. It was not possible to measure the KHN of the tooth surface immediately after caries lesion induction because the substrate was excessively soft. When comparing the KHN values of sound dentine (66.5 ± 3.1) with those obtained caries-affected dentine (25.2 ± 5.4), sound dentine exhibited significantly higher values (*p* < 0.0001; Fig. 1). Notably, the KHN values observed for caries-affected dentine were consistent with those previously reported in the literature (30.75 ± 4.56) [36].

### Microtensile bond strength (µTBS)

The frequency of each fracture pattern is presented in Table 2. The most frequent failure mode was adhesive/mixed failure (97.70%), followed by cohesive failures (1.25%) and premature failures (1.05%) among all specimens (Table 3).

**Table 2:** Weibull modulus, characteristic strength, bond strength and number of specimens, as well as fracture mode according to dentine substrate, adhesive systems, bonding strategy, and treatment

Dentine	Adhesive	Bonding strategy	Treatment	m (95% CI)	σ_0_ (95% CI)	µTBS (MPa)	Failure mode*
Sound	ZipBond Universal	Etch-and-rinse	Untreated	8.3 (6.2–10.5) c	42.0 (31.4–52.6) cd	39.8 ± 5.1 b	[0/60/0/60]
			DMSO	12.2 (9.1–15.3) b	46.2 (34.5–58.0) b	44.1 ± 4.1 ab	[1/59/0/60]
		Self-etch	Untreated	21.4 (16.0–26.9) a	37.3 (27.9–46.8) e	36.6 ± 2.7 b	[2/58/0/60]
			DMSO	14.9 (11.1–18.7) b	42.2 (31.5–52.9) d	40.6 ± 3.1 b	[1/58/1/60]
	Tetric N-Bond Universal	Etch-and-rinse	Untreated	9.7 (7.2–12.2) c	42.7 (31.9–53.5) c	40.4 ± 4.8 b	[0/60/0/60]
			DMSO	10.5 (7.8–13.2) c	54.03 (40.3–67.7) a	51.3 ± 4.4 a	[0/60/0/60]
		Self-etch	Untreated	10.0 (7.4–12.5) c	41.7 (31.2–52.3) d	39.2 ± 4.4 b	[3/56/1/60]
			DMSO	11.8 (8.8–14.8) b	52.9 (39.5–66.3) a	50.5 ± 4.6 a	[1/59/0/60]
Caries-affected	ZipBond Universal	Etch-and-rinse	Untreated	8.7 (6.5–11.0) c	27.2 (20.3–34.2) g	25.5 ± 3.9 c	[2/58/0/60]
			DMSO	12.0 (9.0–15.1) b	42.9 (32.0–53.8) c	40.7 ± 3.9 b	[0/60/0/60]
		Self-etch	Untreated	9.6 (7.2–12.0) c	23.8 (17.8–29.8) h	22.5 ± 3.2 c	[1/59/0/60]
			DMSO	10.2 (7.6–12.7) c	47.7 (35.6–59.8) b	44.1 ± 4.9 ab	[0/60/0/60]
	Tetric N-Bond Universal	Etch-and-rinse	Untreated	9.9 (7.46–12.5) c	31.2 (23.3–39.1) f	29.3 ± 3.7 c	[0/60/0/60]
			DMSO	14.7 (11.0–18.4) b	46.4 (34.6–58.2) b	44.5 ± 3.8 ab	[1/57/2/60]
		Self-etch	Untreated	10.0 (7.5–12.5) c	30.4 (22.7–38.0) f	28.5 ± 3.6 c	[0/57/3/60]
			DMSO	11.0 (8.2–13.8) c	44.5 (33.2–55.8) c	41.3 ± 4.2 b	[0/58/2/60]

* Number in square brackets represent the number of specimens (resin-dentine sticks) corresponding to each fracture mode classification [(1/2/3/4): (1) cohesive failure; (2) adhesive/mixed failure; (3) pre-test failure and (4) total number of tested specimens]. *Abbreviations: m* Weibull modulus, *σ*_*0*_ characteristic strength, *µTBS* microtensile bond strength, and *CI* confidence interval. Different lowercase letters indicate significant differences (columns) according to the Likelihood Ratio Test (LRT) and chi-square distribution (α = 0.05) for *m and σ*_*0*_ and significant difference in *µTBS* among experimental groups according to the Tukey test (*p* < 0.05)

**Table 3:** ANOVA microtensile bond strength results

	Sum of squares	df	Mean square	F	*p*	η^2^
Dentine	2170.746	1	2170.746	114.65672	< 0.001	0.207
Adhesive	495.102	1	495.102	26.15080	< 0.001	0.047
Application mode	80.106	1	80.106	4.23113	0.052	0.001
Treatment	4565.140	1	4565.140	241.12631	< 0.001	0.436
Dentine * Adhesive	48.118	1	48.118	2.54155	0.114	0.005
Dentine * Application mode	14.472	1	14.472	0.76441	0.384	0.001
Adhesive * Application mode	0.164	1	0.164	0.00866	0.926	0.000
Dentine * Treatment	582.428	1	582.428	30.76331	< 0.001	0.056
Adhesive * Treatment	11.246	1	11.246	0.59398	0.443	0.001
Application mode * Treatment	9.277	1	9.277	0.49002	0.485	0.001
Dentine * Adhesive * Application mode	44.557	1	44.557	2.35345	0.128	0.004
Dentine * Adhesive * Treatment	251.328	1	251.328	13.27491	< 0.001	0.024
Dentine * Application mode * Treatment	6.125	1	6.125	0.32352	0.571	0.001
Adhesive * Application mode * Treatment	36.061	1	36.061	1.90472	0.170	0.003
Dentine * Adhesive * Application mode* Treatment	42.228	1	42.228	2.23045	0.138	0.004
Residuals	2120.448	112	18.933			

The four-way repeated-measures ANOVA revealed that the factors “Dentine” (p < 0.001; η² = 0.20), "Adhesive” (*p* < 0.001; η² = 0.04) and “Treatment” (*p* < 0.001; η² = 0.43), as well as the two-way interaction “Dentine × Treatment” (*p* < 0.001; η² = 0.05) and the three-way interaction “Dentine × Adhesive × Treatment” (*p* < 0.001; η² = 0.02), significantly influenced the µTBS values of the tested universal adhesives (Table 3).

**Fig. 2:**
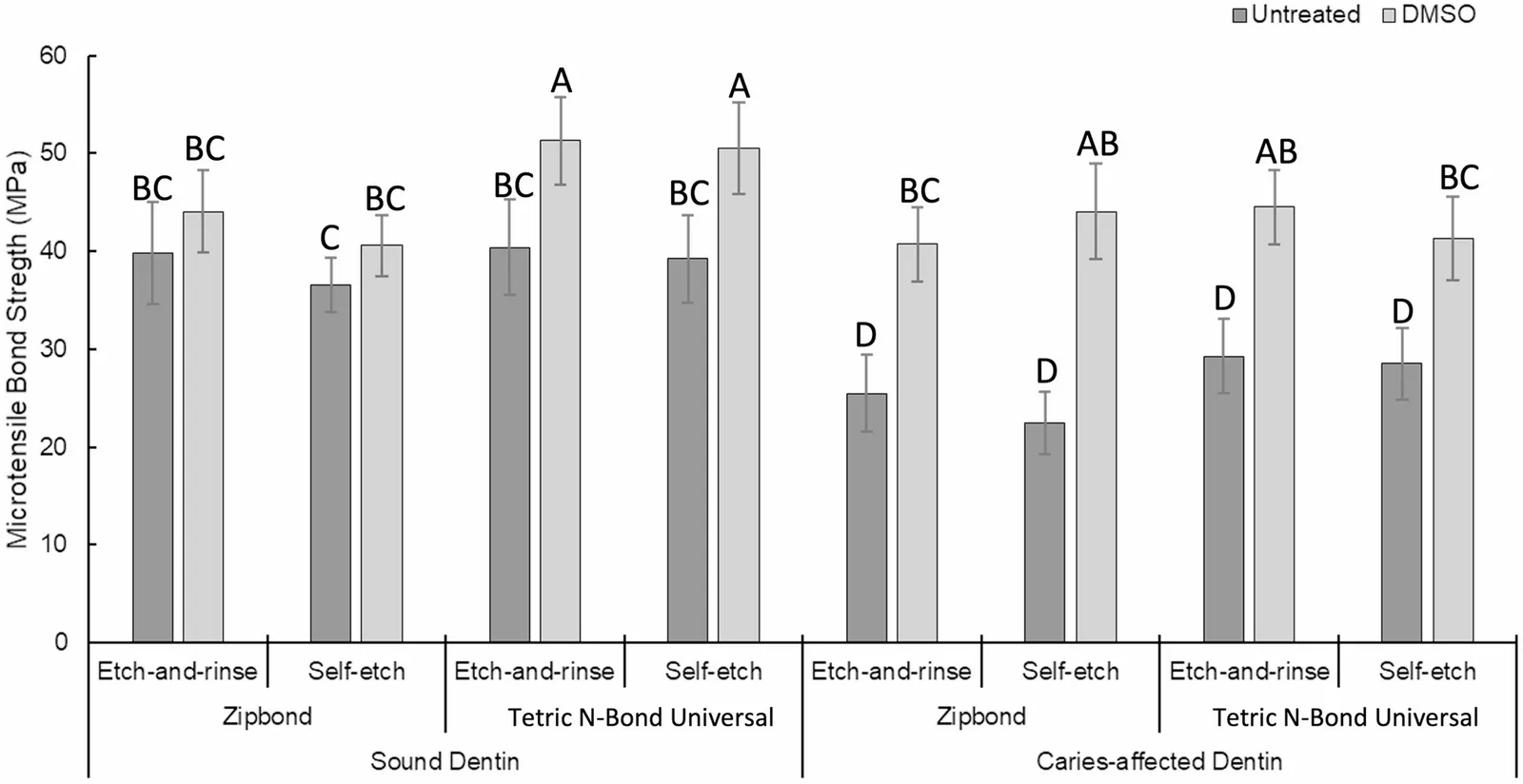
Microtensile bond strength (µTBS) values (MPa; mean ± standard deviation) of universal adhesives ZipBond Universal and Tetric N-Bond Universal using etch-and-rinse or self-etch application modes on sound and caries-affected dentine, either untreated or pretreated with DMSO. Different letters indicate significant differences among all experimental groups (four-way ANOVA; Tukey test; *p* < 0.05)

The mean cross-sectional area of the resin-dentine bonded specimens was 0.82 ± 0.09 mm², ranging from 0.71 to 0.89 mm², with no significant differences among groups regarding specimen size (*p* > 0.05). The µTBS results are presented in Fig. 2 and Table 2.

Overall, sound dentine exhibited significantly higher µTBS values than caries-affected dentine, regardless of the adhesive, treatment, or bonding strategy (*p* < 0.05). DMSO pretreatment promoted a slight increase in bond strength for ZipBond Universal in sound dentine; however, these values were not statistically different from those of the corresponding untreated groups (*p* > 0.05). In contrast, DMSO pretreatment significantly improved the µTBS of Tetric N-Bond Universal in sound dentine for both etch-and-rinse and self-etch strategies, resulting in the highest bond strength values (*p* < 0.05). Among the untreated groups in sound dentine, no significant differences were observed between the etch-and-rinse and self-etch strategies for either adhesive system (*p* > 0.05).

In caries-affected dentine, all untreated groups showed significantly lower µTBS values than their corresponding sound dentine groups (*p* < 0.05). Untreated ZipBond Universal and Tetric N-Bond Universal groups exhibited similar bond strength values, regardless of the bonding strategy employed (*p* > 0.05). After DMSO pretreatment, both adhesive systems demonstrated a significant increase in µTBS in caries-affected dentine (*p* < 0.05). The DMSO-treated groups showed bond strength values statistically comparable to those observed in sound dentine, regardless of the adhesive strategy used (*p* > 0.05). In addition, no significant differences were observed between the etch-and-rinse and self-etch strategies within the caries-affected dentine groups (*p* > 0.05).

**Fig. 3:**
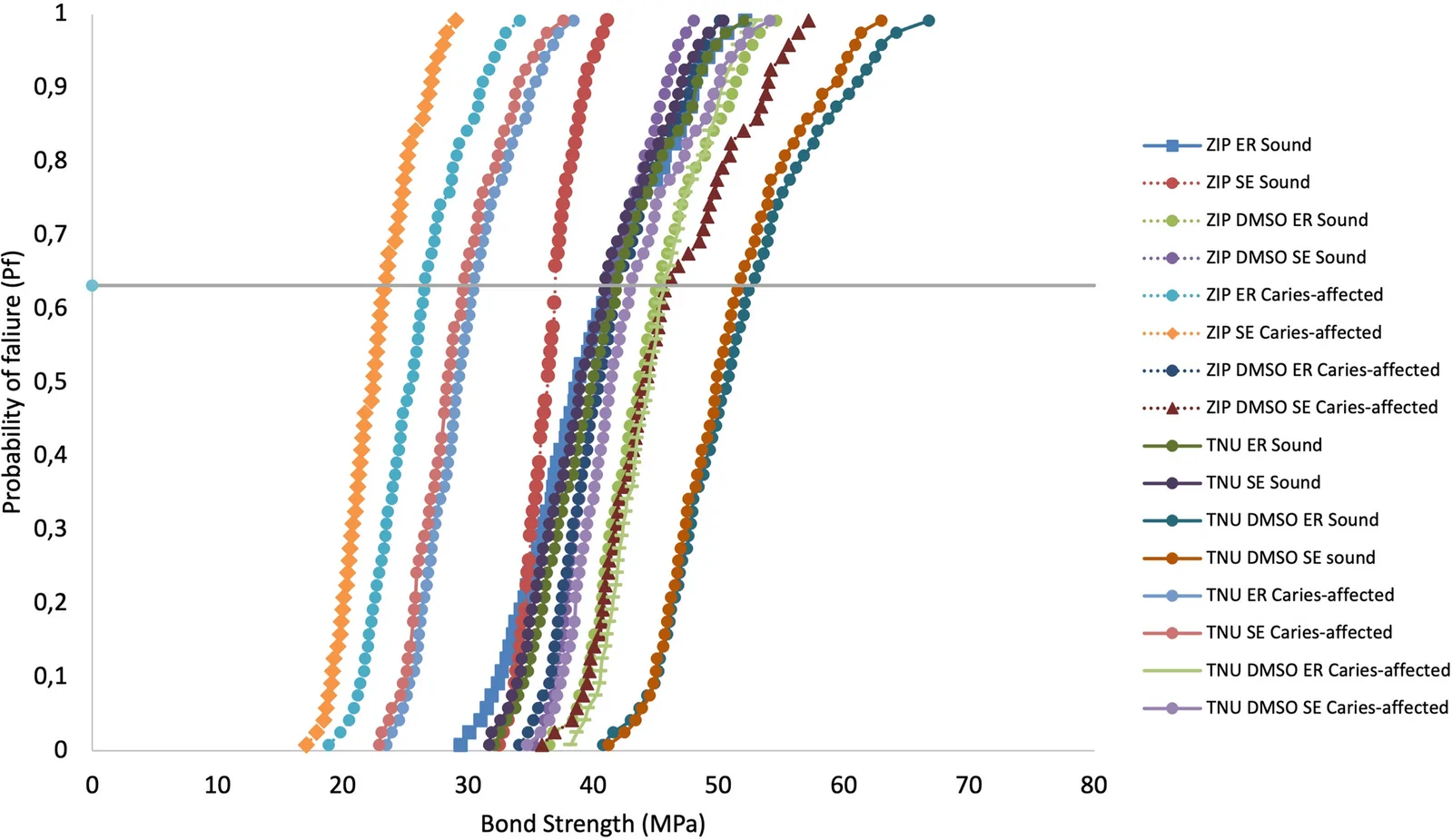
Weibull probability plots of the microtensile bond strength (µTBS) values for ZipBond Universal (ZIP) and Tetric N-Bond Universal (TNU) applied to sound and caries-affected dentine using the etch-and-rinse (ER) and self-etch (SE) strategies, with or without DMSO pretreatment. The horizontal displacement of the curves reflects differences in characteristic strength (σ₀), whereas the slope of the curves represents the Weibull modulus (m), indicating the reliability and variability of the adhesive interface. Curves shifted to the right indicate higher bond strength values, whereas steeper slopes indicate greater bond reliability and lower variability among specimens (pf = 0.632)

The Weibull modulus (m) and characteristic strength (σ₀) values are reported in Table 2, and the corresponding probability-of-failure plots are shown in Fig. 3. Weibull analysis revealed significant differences among the experimental groups for both m and σ₀ (*p* < 0.05). In sound dentine, the highest Weibull modulus was observed for ZipBond Universal applied in self-etch mode without DMSO pretreatment, whereas the remaining groups showed m values ranging from 8.3 to 14.9. In caries-affected dentine, m values ranged from 8.7 to 14.7 and were generally lower than the highest value observed in sound dentine (*p* < 0.05).

Characteristic strength values (σ₀) ranged from 37.3 to 54.0 MPa in sound dentine and from 23.8 to 47.7 MPa in caries-affected dentine. Untreated caries-affected dentine groups exhibited σ₀ values approximately 25–40% lower than those observed in sound dentine. DMSO pretreatment significantly increased σ₀ values for both adhesives and application modes, with increases ranging from approximately 10–13% in sound dentine and 49–100% in caries-affected dentine (*p* < 0.05). The highest σ₀ values were obtained for Tetric N-Bond Universal associated with DMSO pretreatment in sound dentine, whereas the lowest value was observed for untreated caries-affected dentine bonded with ZipBond Universal. In caries-affected dentine, DMSO-treated groups presented σ₀ values comparable to those of most sound dentine groups (*p* > 0.05).

### Nanoleakage evaluation (%)

**Fig. 4:**
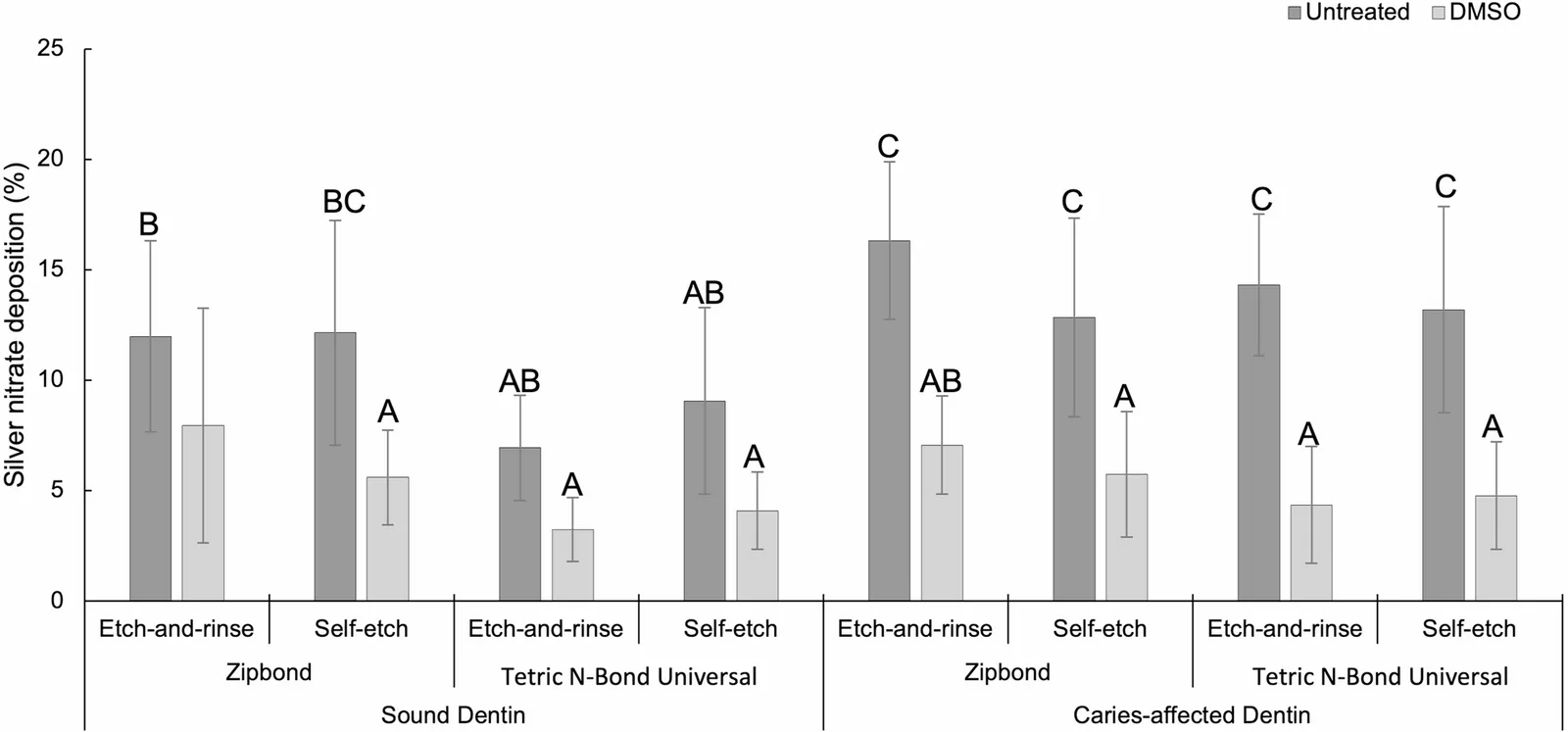
Nanoleakage values (%; mean ± standard deviation) of the universal adhesives Zipbond Universal and Tetric N-Bond Universal in the etch-and-rinse or self-etch application modes to sound and caries-affected dentine, either untreated or pretreated with DMSO. Different letters indicate significant differences among all experimental groups (four-way ANOVA; Tukey test; *p* < 0.05)

**Fig. 5:**
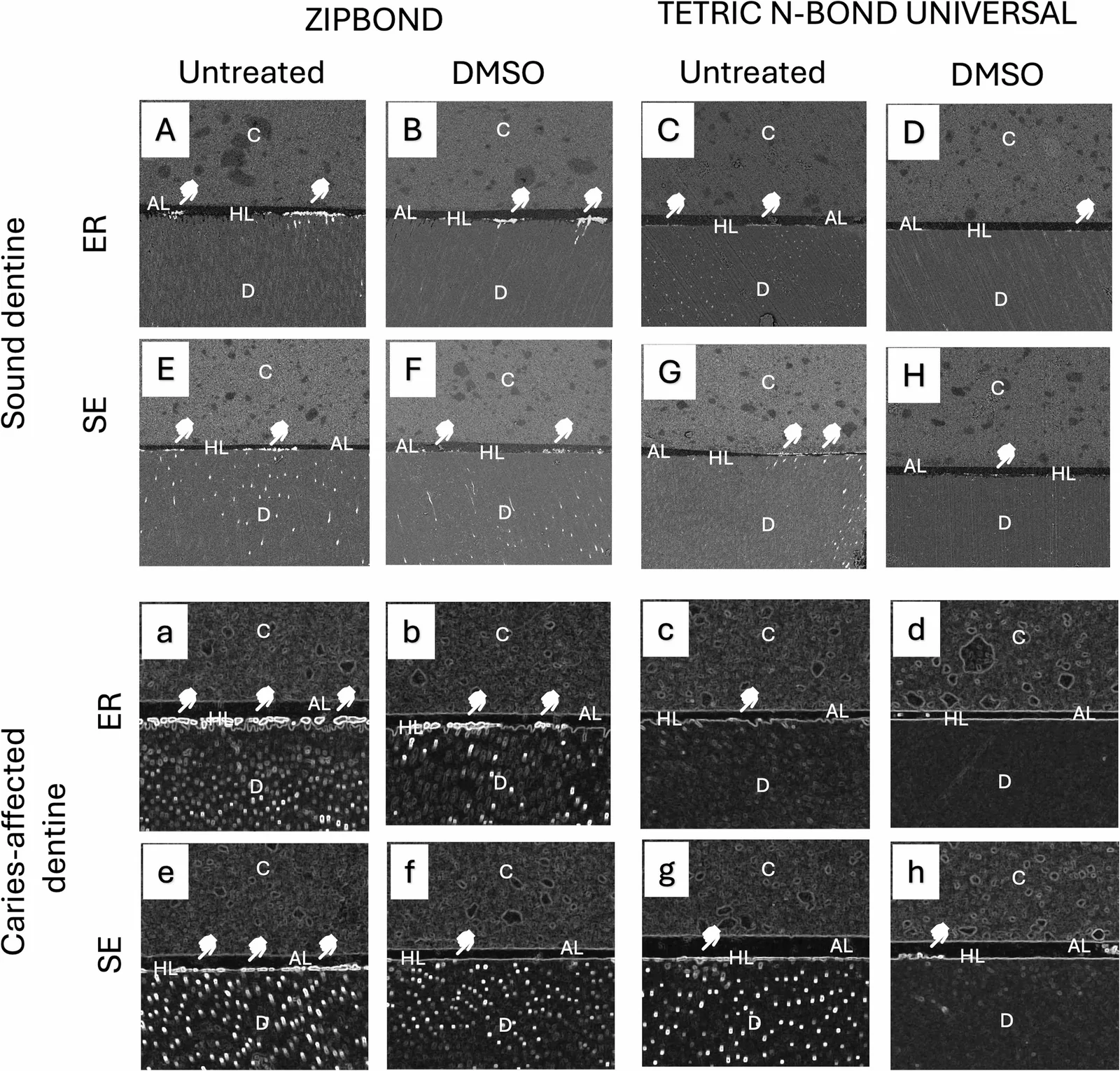
Representative backscattered SEM micrographs (1000x) of the adhesive interfaces produced by the universal adhesives ZipBond Universal and Tetric N-Bond Universal applied in the etch-and-rinse or self-etch application modes to sound (**A**-**F**) and caries-affected dentine (**a**-**f**), either untreated or pretreated with DMSO. Silver deposits were observed along the hybrid layer (white hands) regardless of dentine substrate, adhesive system, treatment or bonding strategy (**A**-**F**; **a**-**f**). In caries-affected dentine, both Zipbond Universal and Tetric N-Bond Universal pretreated with DMSO exhibited lower silver nitrate uptake at the bottom of the hybrid layer than the corresponding untreated groups

The four-way repeated-measures ANOVA revealed that “Dentine” (p = 0.001; η² = 0.04), "Adhesive” (*p* < 0.001; η^2^ = 0.04) and “Treatment” (*p* < 0.001; η^2^ = 0.42), as well as the two-way interaction “Dentine × Treatment” (*p* = 0.003; η² = 0.03), had significant effects on nanoleakage levels of both tested universal adhesives (Table 4). The quantitative nanoleakage data are presented in Fig. 4, whereas representative images of the resin-dentin interface are shown in Fig. 5.

**Table 4:** ANOVA nanoleakage results

	Sum of squares	df	Mean square	F	*p*	η^2^
Dentine	140.3538	1	140.3538	11.21044	0.001	0.043
Adhesive	140.2526	1	140.2526	11.20235	0.001	0.043
Application mode	11.3442	1	11.3442	0.90609	0.343	0.003
Treatment	1364.7620	1	1364.7620	109.00719	< 0.001	0.420
Dentine * Adhesive	59.3314	1	59.3314	4.73895	0.032	0.018
Dentine * Application mode	19.6410	1	19.6410	1.56878	0.213	0.006
Adhesive * Application mode	62.0466	1	62.0466	4.95583	0.028	0.019
Dentine * Treatment	114.6448	1	114.6448	9.15699	0.003	0.035
Adhesive * Treatment	2.3112	1	2.3112	0.18460	0.668	0.001
Application mode * Treatment	0.0432	1	0.0432	0.00345	0.953	0.000
Dentine * Adhesive * Application mode	0.6989	1	0.6989	0.05583	0.814	0.000
Dentine * Adhesive * Treatment	1.1979	1	1.1979	0.09568	0.758	0.000
Dentine * Application mode * Treatment	24.7636	1	24.7636	1.97794	0.163	0.008
Adhesive * Application mode * Treatment	3.9313	1	3.9313	0.31400	0.576	0.001
Dentine * Adhesive * Application mode* Treatment	0.1095	1	0.1095	0.00875	0.926	0.000
Residuals	1302.0723	104	12.5199			

Overall, caries-affected dentine exhibited significantly higher nanoleakage values than sound dentine, regardless of the adhesive or bonding strategy employed (*p* < 0.05). Untreated groups consistently demonstrated the highest nanoleakage levels for both adhesive systems and for both etch-and-rinse and self-etch approaches. In sound dentine, untreated groups showed significantly higher nanoleakage values than their corresponding DMSO-treated groups, with no significant differences between etch-and-rinse and self-etch strategies within the same adhesive system.

DMSO pretreatment significantly reduced nanoleakage values in both dentine substrates (*p* < 0.05). In sound dentine, DMSO-treated groups exhibited lower silver nitrate deposition than the corresponding untreated groups, irrespective of the adhesive system or bonding strategy. A similar trend was observed in caries-affected dentine, where DMSO pretreatment also significantly reduced nanoleakage values for both ZipBond Universal and Tetric N-Bond Universal (*p* < 0.05).

Among all experimental conditions, untreated caries-affected dentine groups exhibited the highest nanoleakage values (*p* < 0.05), indicating greater interfacial permeability and poorer sealing ability of the adhesive interface. Conversely, DMSO-treated caries-affected dentine groups showed significantly reduced nanoleakage levels that were comparable to those observed in several untreated sound dentine groups (*p* > 0.05).

When adhesive systems and bonding strategies were compared, both universal adhesives exhibited similar nanoleakage behavior. No significant differences were observed between ZipBond Universal and Tetric N-Bond Universal under comparable experimental conditions (*p* > 0.05). Likewise, etch-and-rinse and self-etch strategies generally resulted in similar nanoleakage values within each adhesive system, with only minor variations among some DMSO-treated groups (*p* > 0.05).

### Degree of conversion

**Fig. 6:**
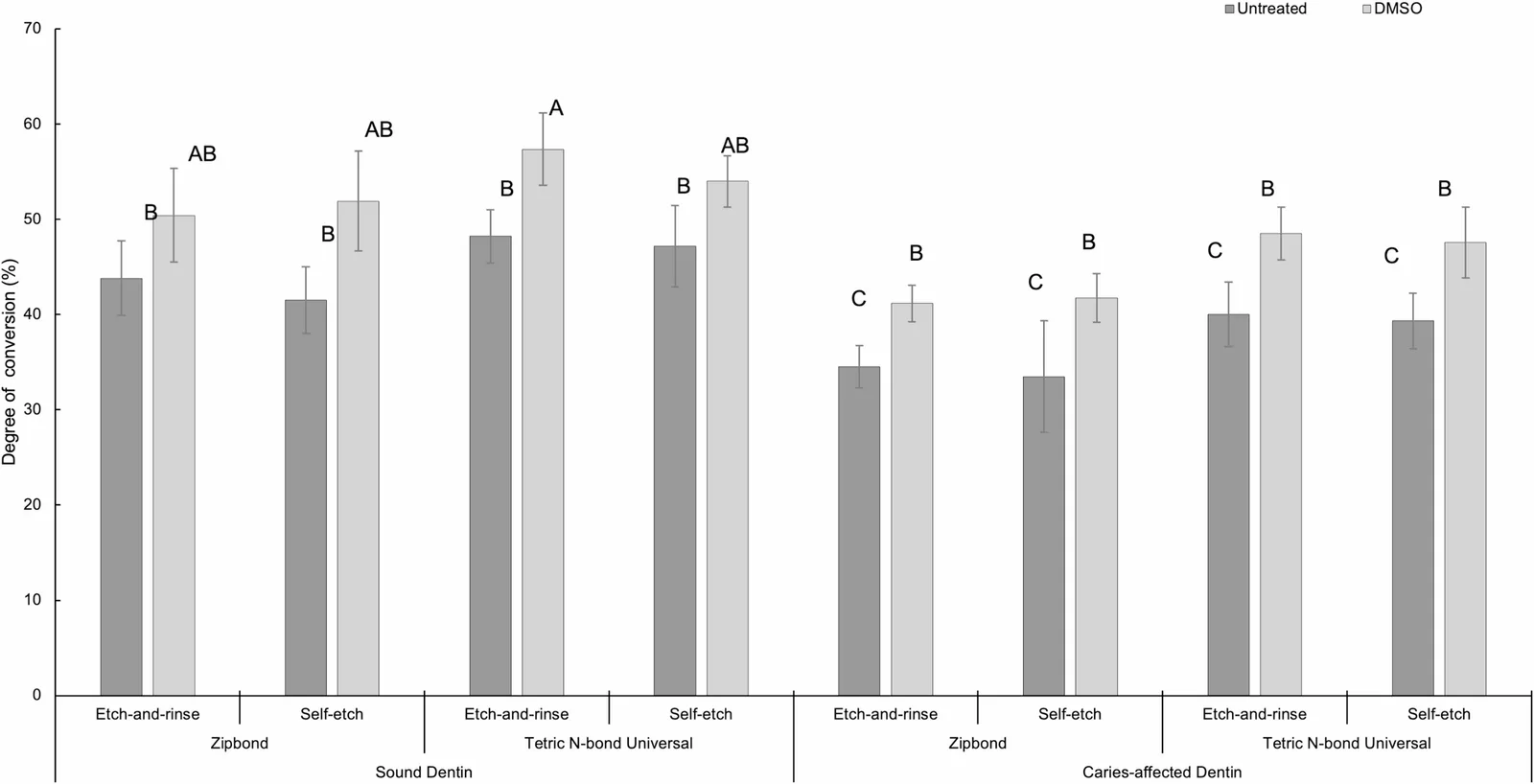
Degree of conversion values (%; mean ± standard deviation) of the universal adhesives ZipBond Universal and Tetric N-Bond Universal applied in the etch-and-rinse or self-etch application modes to sound and caries-affected dentine, either untreated and pretreated with DMSO. Different letters indicate significant differences among all experimental groups (four-way ANOVA; Tukey test; *p* < 0.05

The four-way repeated-measures ANOVA revealed that both “Dentine” (p < 0.001; η² = 0.33), "Adhesive” (*p* < 0.001; η² = 0.138) and “Treatment” (*p* < 0.001; η² = 0.30) had significant effects on the degree of conversion of the tested universal adhesives (Table 5). Degree of conversion values (%) are reported in Fig. 6.

**Table 5:** ANOVA degree of conversion results

	Sum of squares	df	Mean square	F	*p*	η^2^
Dentine	1740.29570	1	1740.29570	131.5128	< 0.001	0.337
Adhesive	714.16860	1	714.16860	53.9692	< 0.001	0.138
Application mode	20.00200	1	20.00200	1.5115	0.223	0.004
Treatment	1566.55042	1	1566.55042	118.3830	< 0.001	0.303
Dentine * Adhesive	11.38504	1	11.38504	0.8604	0.356	0.002
Dentine * Application mode	3.54202	1	3.54202	0.2677	0.606	0.001
Adhesive * Application mode	8.70010	1	8.70010	0.6575	0.420	0.002
Dentine * Treatment	0.67000	1	0.67000	0.0506	0.823	0.000
Adhesive * Treatment	0.25627	1	0.25627	0.0194	0.890	0.000
Application mode * Treatment	2.86350	1	2.86350	0.2164	0.643	0.001
Dentine * Adhesive * Application mode	2.36882	1	2.36882	0.1790	0.673	0.000
Dentine * Adhesive * Treatment	3.21934	1	3.21934	0.2433	0.623	0.001
Dentine * Application mode * Treatment	0.00667	1	0.00667	0.000504	0.982	0.000
Adhesive * Application mode * Treatment	23.58184	1	23.58184	1.7821	0.186	0.005
Dentine * Adhesive * Application mode* Treatment	6.80535	1	6.80535	0.5143	0.475	0.001
Residuals	1058.63167	80	13.23290			

Overall, sound dentine showed higher degree of conversion values than caries-affected dentine (*p* < 0.05). In sound dentine, DMSO pretreatment significantly increased the degree of conversion compared with the corresponding untreated groups (*p* < 0.05). The highest degree of conversion values were observed in the DMSO-treated groups, whereas lower values were recorded for the corresponding untreated groups.

In caries-affected dentine, untreated groups exhibited the lowest degree of conversion among all experimental conditions. DMSO pretreatment significantly increased the degree of conversion values for both adhesive systems and bonding strategies (*p* < 0.05), with improvements ranging from approximately 8 to 10% compared with the corresponding untreated groups. Although DMSO pretreatment markedly improved the degree of conversion in caries-affected dentine, the values remained lower than those observed for the highest-performing sound dentine groups. However, some DMSO-treated caries-affected dentine groups became statistically comparable to untreated sound dentine groups (*p* > 0.05).

## Discussion

The structural and compositional alterations of caries-affected dentine make adhesion to this substrate more challenging than adhesion to sound dentine [31, 44]. In addition, the considerable variability in lesion characteristics, including lesion depth, dentine hardness, caries activity, patient age, and anatomical location, makes reproducing natural dentinal caries under laboratory conditions particularly difficult [12, 44]. Therefore, the present study aimed to standardize the artificial lesions while reproducing the key structural and morphological features of naturally occurring caries-affected dentine. For this purpose, a microbiological model was selected because of its ability to reproduce clinically relevant characteristics of dentinal caries, including discoloration, bacterial contamination, demineralization, and collagen alterations similar to those observed in natural lesions [12, 36]. Such models have been extensively used in bonding studies involving caries-affected dentine because they provide greater biological relevance and substrate reproducibility [13, 45–47].

In the present study, DMSO pretreatment was applied to both sound and caries-affected dentine prior to the use of two universal adhesives in both etch-and-rinse and self-etch application modes. The use of DMSO as a dentine pretreatment has been extensively investigated in sound dentine because of its ability to improve adhesive infiltration into the demineralized collagen matrix [26, 30, 48]. Due to its amphiphilic nature, DMSO interacts with both hydrophilic and hydrophobic components, increasing dentine wettability and modifying collagen matrix dynamics [23]. These effects may facilitate monomer diffusion throughout the collagen scaffold, thereby enhancing adhesive infiltration [23]. Previous studies have demonstrated improved bonding performance following DMSO pretreatment in sound dentine [30, 49–52]. Although lower DMSO concentrations have shown promising results in previous studies [53], 50% DMSO was selected based on the evidence available from sound dentine. Its effectiveness in caries-affected dentine, however, had not been established, warranting its investigation in this distinct substrate.

However, in the present study, the beneficial effect of DMSO on sound dentine was not consistent across all experimental conditions. For ZipBond Universal, DMSO pretreatment did not improve bond strength. This finding may be related to the specific physicochemical characteristics of this adhesive system. ZipBond Universal is an ultra-mild, HEMA-free universal adhesive containing 10-MDP and ethanol as its solvent [53, 54], and its formulation was designed to promote effective dentine infiltration even in the absence of HEMA. The bonding behavior of HEMA-free adhesives is known to be highly material-dependent and influenced by factors such as solvent composition, hydrophilicity, viscosity, and monomer interactions [53, 54]. Moreover, the absence of HEMA may alter solvent evaporation dynamics and increase the susceptibility of simplified adhesive formulations to phase separation [55, 56]. Because the effects of DMSO are closely associated with enhanced dentine wettability and improved resin monomer diffusion within the collagen matrix [48], its influence may also depend on the chemical composition and solvent characteristics of the adhesive system [53]. One possible explanation is that the formulation of ZipBond Universal may have influenced its response to DMSO pretreatment in sound dentine. However, the present findings do not establish the specific physicochemical mechanisms underlying this response.

Besides the findings observed in sound dentine, the main finding of the present study was that DMSO pretreatment improved the bonding performance of both universal adhesives in caries-affected dentine. Although its benefits in sound dentine depended on the adhesive system, DMSO improved µTBS in caries-affected dentine and was associated with reduced nanoleakage and increased degree of conversion. These findings suggest that DMSO may help overcome some of the challenges associated with bonding to structurally compromised dentine. However, its effects were not uniform across all outcomes. Improvements in bond strength and interfacial sealing appeared to depend on the dentine substrate and adhesive-related characteristics, whereas the increase in degree of conversion was observed in both sound and caries-affected dentine. Taken together, these findings support the partial rejection of all three null hypotheses and suggest that the benefits of DMSO may involve more than improved polymerization alone, potentially including enhanced resin infiltration and improved sealing of the dentine substrate.

The reduced bonding effectiveness to caries-affected dentine is closely related to the structural and chemical alterations resulting from the caries process [7, 12]. Partial demineralization and collagen degradation increase dentine porosity and intrinsic water content, creating a substrate that is less receptive to hydrophobic methacrylate monomers [57, 58]. In addition, disruption of the collagen network and loss of interfibrillar integrity compromise adequate resin infiltration and favor the formation of defective hybrid layers [59, 60]. Under these conditions, the presence of residual water within the collagen matrix becomes particularly detrimental because it interferes with solvent evaporation, monomer diffusion, and polymerization kinetics at the adhesive interface [56, 61].

Within this context, the amphiphilic nature of DMSO may play a particularly relevant role. Because DMSO interacts with both water and organic components, it may improve the compatibility between the intrinsically moist dentine substrate and the hydrophobic components of adhesive systems [23, 48]. Previous studies have shown that DMSO modifies collagen matrix dynamics, improves dentine wettability, and increases interfibrillar spacing, thereby facilitating monomer diffusion throughout demineralized dentine [62]. These effects may be especially relevant in caries-affected dentine, where increased water content and greater exposure of collagen fibrils can complicate the formation of a stable adhesive interface [12]. By modifying the dentine environment, DMSO may facilitate resin infiltration while also creating conditions more favorable for monomer polymerization. However, its influence on polymerization is unlikely to be restricted to caries-affected dentine, suggesting that this aspect of its action may reflect a more general effect on the bonding substrate rather than a response specific to caries-related alterations.

Furthermore, the increased degree of conversion observed after DMSO pretreatment may also be related to its ability to reduce residual water within the bonding substrate. Excess water is known to interfere with free-radical polymerization, promote phase separation [56], and impair monomer conversion in simplified adhesive systems [40]. By improving substrate wettability while reducing the detrimental effects of excess water, DMSO may have provided a more favorable environment for polymer network formation [23]. This mechanism may be particularly relevant in caries-affected dentine, where the higher intrinsic water content has been associated with reduced polymerization efficiency and compromised adhesive performance [31]. Although this mechanism may contribute to the improvements in degree of conversion observed after DMSO pretreatment, the present results do not demonstrate that it produces a greater effect in caries-affected dentine than in sound dentine.

The Weibull analysis further supports these findings. In addition to increasing bond strength values, DMSO pretreatment substantially increased the characteristic strength (σ₀) of caries-affected dentine for both universal adhesives and bonding strategies. Because σ₀ corresponds to the stress level at which approximately 63.2% of the specimens are expected to fail, higher values are generally considered indicative of greater structural integrity and mechanical reliability of the adhesive interface [63]. From a fracture mechanics perspective, these findings suggest that DMSO pretreatment may have reduced the presence of critical defects and stress concentration areas commonly associated with poorly infiltrated hybrid layers. Moreover, the higher Weibull modulus (*m*) values observed in some DMSO-treated groups indicate lower variability and a more homogeneous distribution of failure stresses within the adhesive interface. These findings are also consistent with the lower nanoleakage values observed after DMSO pretreatment [30], supporting the hypothesis that DMSO may have promoted more uniform monomer infiltration and polymer network formation within the porous collagen matrix of caries-affected dentine.

Interestingly, the beneficial effect of DMSO on caries-affected dentine was also observed with the HEMA-free adhesive, which did not show improved bonding performance in sound dentine. In caries-affected dentine, the unfavorable substrate characteristics may become more influential than differences related solely to adhesive composition. The greater structural heterogeneity of this substrate increases the susceptibility of the adhesive interface to limitations associated with solvent evaporation, monomer diffusion, and incomplete collagen encapsulation [7, 12, 61]. Under these conditions, the ability of DMSO to enhance dentine wettability and improve the interaction between hydrophilic and hydrophobic phases may partially offset compositional differences among adhesive systems [23], enabling adhesives with distinct hydrophilicity profiles and solvent compositions to benefit from DMSO pretreatment.

Furthermore, the interaction between DMSO and 10-MDP, the functional monomer present in both universal adhesives evaluated in the present study, may also have contributed to the improved bonding performance observed in caries-affected dentine. This substrate is characterized by partial demineralization, increased porosity, and a higher water content than sound dentine, conditions that may compromise monomer infiltration and the establishment of stable adhesive interfaces. The 10-MDP monomer is known to chemically interact with residual hydroxyapatite through ionic bonding with calcium, leading to the formation of stable and insoluble MDP–Ca salts within the hybrid layer. In addition, 10-MDP may establish relatively stable hydrophobic interactions with collagen through its hydrophobic moieties, further contributing to effective dentine bonding [64].

However, in caries-affected dentine, collagen fibrils are surrounded by both bound and unbound water because of the altered mineral and organic composition of the substrate [65]. This excess water may hinder the interaction between functional monomers and collagen, thereby limiting adhesive infiltration and hybrid layer formation [65]. DMSO may also help overcome these limitations. Its methyl groups confer hydrophobic characteristics, whereas the oxygen atom can form hydrogen bonds with water molecules, disrupting water self-association. By modifying the organization of water within the demineralized collagen matrix, DMSO may reduce the amount of unbound water and expose additional collagen-binding sites [66]. This effect may be particularly relevant in caries-affected dentine, where the highly hydrated collagen network represents a major challenge for effective resin infiltration. Consequently, DMSO may facilitate closer interaction between 10-MDP and collagen fibrils while also enhancing monomer diffusion throughout the altered dentine matrix [67]. Collectively, these mechanisms may partly explain the improved bonding performance observed in caries-affected dentine following DMSO pretreatment.

Some limitations of the present study should be considered when interpreting the findings. First, despite the microbiological model reproducing several important characteristics of natural dentinal caries, artificial lesions cannot fully replicate the biological and structural complexity of clinically occurring lesions. In addition, the evaluation focused primarily on immediate bonding performance; therefore, the durability of DMSO-treated interfaces under aging conditions remains unclear. Only two universal adhesives were evaluated, limiting the extrapolation of these findings to other adhesive formulations with different solvent systems, functional monomers, or hydrophilicity profiles. Furthermore, although DMSO pretreatment improved bond strength, degree of conversion, nanoleakage, and bonding reliability, additional investigations evaluating enzymatic degradation, long-term interfacial stability, and clinical performance are required to confirm the durability and clinical relevance of these findings.

## Conclusion

DMSO pretreatment positively affected the resin-bonding performance to caries-affected dentine, regardless of the universal adhesive system or bonding strategy employed. Improvements in bond strength were accompanied by a higher degree of conversion, greater bonding reliability, and reduced nanoleakage, suggesting an overall improvement in adhesive interface quality. Although the effect of DMSO on sound dentine appeared to be material-dependent, its benefits were more pronounced in structurally compromised substrates. Therefore, DMSO pretreatment appears to be a promising strategy for improving the quality and reliability of resin-dentine bonding to caries-affected dentine.

## Data Availability

The datasets generated and/or analyzed during the current study are available from the corresponding author upon reasonable request.
